# Correction: Catecholamines reduce choice history biases in perceptual decision making

**DOI:** 10.1371/journal.pbio.3003548

**Published:** 2025-12-05

**Authors:** Jan Willem de Gee, Niels A. Kloosterman, Anke Braun, Tobias H Donner

In Fig 2, the panel E is incorrect. The “-0.5” and “0” labels on the y-axis should be “-0.05” and “0.00”. Please see the correct Fig 2 here.

**Fig 2:**
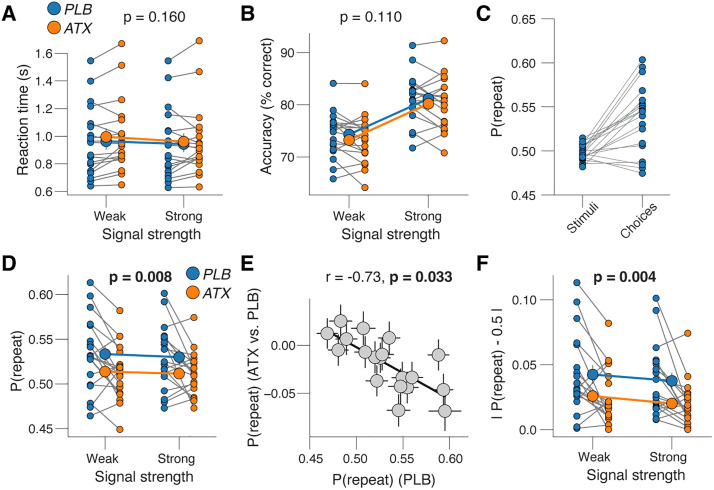
Atomoxetine reduces choice history bias. **(A)** Reaction time, separately for placebo and atomoxetine sessions, and separately or weak and strong signals. Every connecting line is a participant; large data points in the middle are the group averages (error bars, s.e.m. across 19 participants); stats, main effect of drug in two-way repeated measures ANOVA. See S2A Fig for reaction time distributions. **(B)** As A, but for accuracy. **(C)** Probability of repeating the previous stimulus category (left) or choice (right) during placebo sessions. **(D)** As A, but for repetition probability (Materials and methods). **(E)** Individual shift in repetition probability caused by atomoxetine, plotted against individual’s repetition probability during the placebo sessions. Data points are individual participants; error bars, 68% confidence intervals across 5K bootstraps; stats, Pearson’s correlation coefficient (corrected for reversion to the mean; S2E Fig; Materials and methods). **(F)** As A, but for absolute repetition probability (Materials and methods).

The ORCID iDs are missing for the second, third, and fourth author. Please see the authors’ respective ORCID iDs here:

- Author Niels A. Kloosterman’s ORCID iD is: 0000-0002-1134-7996
  (https://orcid.org/0000-0002-1134-7996).
- Author Anke Braun’s ORCID iD is: 0000-0002-1946-7765
  (https://orcid.org/0000-0002-1946-7765).
- Author Tobias H. Donner’s ORCID iD is 0000-0002-7559-6019
  (https://orcid.org/0000-0002-7559-6019).
