# Supplementary material for: Catecholamines reduce choice history biases in perceptual decision making
Source: PLoS Biol. 2025 Sep 3;23(9):e3003361. doi: 10.1371/journal.pbio.3003361 (PMC12407465; doi:10.1371/journal.pbio.3003361)
Supplement: Attachment — Submitted filename: deGeeEtAlRebuttal_final.pdf [file pbio.3003361.s007.pdf]

REVIEWS:

Reviewer #1 (Rei Akaishi):

The authors have conducted an exemplary study examining catecholamine effects on choice behavior, specifically focusing on tendencies to either repeat or alternate choices. This well-written paper addresses a topic of central importance to cognitive neuroscience. The study involved administering atomoxetine to subjects before they performed standard perceptual decision-making tasks with two visual stimuli. The study's conception is well-grounded in theoretical ideas about catecholamines and previously observed effects of arousal on choice repetition tendencies.

Thank you for the overall positive assessment of our work.

While the authors' ideas appear clear initially, there are several alternative interpretations of the results and some inconsistencies between current and previous theoretical frameworks. Additionally, there are some conceptual ambiguities and interpretational issues within the current study. The authors should address these concerns to strengthen the paper.

Thank you for your thoughtful input, which helped us to improve the manuscript.

Major Issues:

1. While the authors attribute atomoxetine's effects on choice repetition/alternation to its central action on the brain's norepinephrine system, the orally administered drug also affects peripheral systems. Indeed, the authors report acute effects on heart rate. This raises the possibility that the observed behavioral effects stem from peripheral changes that subsequently influence central information processing. The authors should examine relationships between peripheral effects (heart rate and pupil size) and choice tendencies, although control analyses were performed for RT and accuracy. Additional measures of cognitive function would help establish the drug's central effects, particularly given that atomoxetine's acute effects are predominantly peripheral.

Thank you for raising this important point, which we have addressed in three ways:

First, we added the following text to the Introduction (lines 74-78):

> "The administration of atomoxetine at the dosage used in the current study (40 mg) produces robust central effects on several neurophysiological markers of the state of cortical networks: suppression of low-frequency spectral power[1] as well as changes in long-range temporal correlations of local amplitude envelopes[1], in inter-area correlations during task[2] and in signatures of evidence accumulation[3,4]."

Second, we performed a new analysis and added the following text to the Results (lines 106-111):

> "Pupil size is a measure of central arousal[5]. Non luminance-mediated changes of pupil diameter are causally related to the activity of neuromodulatory centers in the brainstem, including the noradrenergic locus coeruleus[6–9]. There was no statistically significant correlation between the individual drug effects on pupil size and on heart rate (**Figure S1B**), in line with the idea that pupil size reflects central arousal more closely than heart rate, a measure of peripheral arousal."

Third, we have added a systematic characterization of the relationship between pupil responses and choice tendencies (during placebo) to the Results (lines 207-240). In short, we found that (i) the magnitude of task-evoked pupil responses during placebo predicted a reduction of repetition probability, mimicking the effect of atomoxetine (**Figure 4A,B**), and (ii) across-subjects, the drug effect on pupil size predicted the drug effect on repetition probability (**Figure 4C)**.

Taken together, we believe that the combination of these findings convincingly demonstrates atomoxetine's effect on central arousal.

2. Assuming central effects are present, there is a theoretical discrepancy between this study and Dayan and Yu's previous work on catecholamine effects in the central nervous system. The Dayan-Yu

framework focuses not on norepinephrine's repetition effects per se, but on cue relevance among multiple cues. This conceptual difference requires explanation.

Thank you. Indeed they illustrate their framework with the “Extended Posner Task”, that involves (differently colored) cues[10]. Crucially, their task involves “state changes”, after which previously learned cue-target relationships are no longer valid. Their explicit hypothesis about the role of noradrenaline (NE) is: “enhancing NE results in greater unexpected uncertainty and therefore a greater readiness to abandon the current hypothesis and adopt a new model for environmental contingencies”.

Even though our task lacks any correlations between stimuli presented on successive trials (**Figure 2C**; the standard in psychophysical laboratory experiments), participants still developed choice history biases (**Figure 2C**), as found in many previous studies including your own[11]. This bias likely results from their (implicit) hypothesis that the world is relatively stable, i.e., that stimuli presented on successive trials are correlated. Thus, our finding that atomoxetine lowers this bias is in line with the Dayan-Yu framework. We have now addressed this more explicitly in the Discussion (lines 265-268).

3. The task design is relevant here, as cue-based attention could select one of the Gabor patches and sustain choice repetition. Thus, the observed drug effects may reflect attentional processes in stimulus selection rather than purely decision processes.

In the absence of direct manipulations and measurements of selective attention, we are agnostic with respect to its role in our current task. Specifically, we have used a principled decision-theoretic framework to quantify and model behavior, which does not make assumptions about the role of attention in the latent computations.

That said, our results, especially the effect of atomoxetine on DDM drift bias, are perfectly consistent with the idea that "attention selects one of the Gabor patches for several subsequent trials and thereby produces choice repetition" and "that the observed drug effects may reflect a reduced repetition effect in attentional selection”. We have added a Discussion paragraph along (lines 268-270).

4. There are concerns about the study's hypotheses and testing methods. Hypothesis (ii) appears ad-hoc. The authors should clarify whether these hypotheses were preregistered.

Our stated hypothesis was: “Specifically, we hypothesized that catecholamines down-regulate the impact of previous experiences on decision-making, which should translate into a reduction of choice history bias.” This hypothesis was not pre-registered, but followed directly from computational theory[10] and earlier correlational work[12]. See also our reply to R1 point 2.

5. The support for hypothesis (ii) in Figure 2E is somewhat weak, as the strengthening of alternation by atomoxetine is not clearly demonstrated. While data show that stronger repetition biases are reduced toward 0.5 choice probability with atomoxetine, the statement about correlation between placebo session biases and atomoxetine-induced shifts requires stronger evidence.

Agreed. We have toned down this conclusion. In the Results we now write (lines 142-144):

> “Further, our data are consistent with the idea that atomoxetine also reduced alternation biases, although the latter were only observed in five participants and weak (**Figure S2E**, data points on x-axis < 0.5).”

In the Discussion we now write (lines 256-258):

> “Using selective pharmacological interventions in humans, we here imply catecholamines in the modulation of human choice history biases: boosting catecholamines levels through atomoxetine specifically reduced the magnitude of choice history biases.”

6. The use of the drift diffusion model to examine atomoxetine's effect on choice repetition appears inconsistent with the authors' earlier support for hypothesis (ii). Hypothesis (ii) addresses both repetition and alternation, yet these separate analyses yield seemingly contradictory results that need reconciliation.

Since most participants exhibited a choice repetition bias, and we no longer claim a drug effect on alternation bias, we deem it appropriate to directly compare starting point or drift bias between atomoxetine and placebo conditions (**Figure 3B**). Note that here it is problematic to take absolute values for starting point and drift bias, because these parameters often have opposing effects[13] (see also our reply to R2 point 2). Finally, in **Figure 3C** we present the relevant individual differences analysis. See also our reply to R1 point 5.

Reviewer #2:

This study investigates the effect of catecholamines on perceptual choice repetition biases. The authors found that administering the noradrenaline reuptake inhibitor atomoxetine reduced choice history biases compared to placebo. Further, by fitting drift diffusion models the authors found that this was due to a reduction in history-dependent shifts of drift rate rather than the starting point of evidence accumulation. Together, the results are taken as evidence that catecholamines reduce the impact of a specific form of priors (choice history) on perceptual decisions. The study addresses a timely topic of perceptual choice history biases and contributes to our understanding of the role of neuromodulatory systems in perceptual decision-making. The methodology appears sound, the data carefully analyzed, and the manuscript well written.

Thank you for the overall positive assessment of our work.

I have a few comments that will hopefully help to improve the manuscript, but otherwise I think it will be a great addition to the literature.

Thank you for your thoughtful input, which certainly helped us to improve the manuscript. See our detailed responses below.

Major comment:

1. The authors quite strongly emphasize the point that atomoxetine reduces choice history biases *regardless of whether those promoted choice repetition or alternation*. However, 15 out of 19 participants exhibit choice repetition, and the remaining 4 participants' repetition probability is fairly close to 50%. In other words, there are no prominent alternation biases in the current data. I understand that the correlation in Figure 2E would imply that if there were participants with pronounced alternation biases, as observed previously, they would be expected to show a reduction towards 50%. I think this extrapolation would be a fair assumption to make, but this assumption should be made more explicit in the Results and Methods.

Agreed. We have toned down this conclusion. See also our reply to R1 point 5.

Minor comments:

2. The authors show that atomoxetine reduces the history shift in drift bias (Fig. 3B). From the analyses it is not entirely clear whether this is a reduction towards zero shift, or a change towards opposite (alternating) shifts. The strong correlation between the effect of drug on P(repeat) and drift bias (Fig. 3C) together with the analyses of P(repeat) in Fig. 2E would suggest that the former is the case, but the authors could repeat their correlation analysis (Fig. 2E) for drift to directly show that participants with larger drift biases in the Placebo condition show the strongest reduction towards zero drift bias in the drug condition.

Thank you for this interesting suggestion. We plotted the correlations for the history shifts in starting point and drift bias (**Figure R1**). Neither correlation is significant when correcting for reversion to the mean (as in **Figure 2E**; Methods), suggesting that the interplay between drift bias and starting point might underlie the effects in P(repeat), rather than only drift bias. These plots also show that the starting point was shifted away from the previous response for most participants (negative values along x-axis in **Figure R1A**) and drift bias was shifted towards the previous response (positive values along x-axis in **Figure R1B**). These opposing effects have been reported before[13] and could explain the non-significant results here.

We added the following text to the Results (lines 188-191):

"Across subjects, the history-dependent shift in starting point did not predict a drug effect therein (r = -0.23, p = 0.684; corrected for reversion to the mean), and the same was true for shifts in drift bias (r = -0.61, p = 0.262),"

We also added the following text to the legend of **Figure S3** (lines 708-711):

> *"The plots show that the starting point was shifted away from the previous response for most participants (negative values along x-axis in left panel) and drift bias was shifted towards the previous response (positive values along x-axis in right panel. These opposing effects have been reported before[13]."*

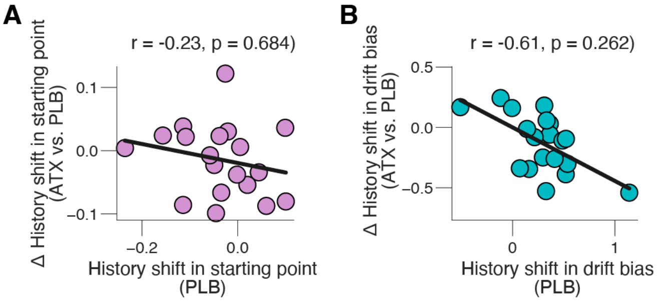


***Figure R1.*** ***(A)*** *Individual shift in starting point caused by atomoxetine, plotted against individual's starting point during the placebo sessions. Data points are individual participants; stats, Pearson's correlation coefficient (corrected for reversion to the mean; Methods).* ***(B)*** *As A, but for drift bias.*

3. Have the authors looked at the effect of drug on choice repetition after correct and error trials? This would be particularly relevant when discussing differences between the current results and previous results involving pupil size. In a previous paper, it was found that the interaction between pupil size and choice repetition was opposite following correct and error trials, and resulted in a net repulsion bias (Supplementary Fig. 11, Urai et al., 2017). Is a similar gating by previous outcome present in the current data, perhaps with a dominance of post-error correction that reduces choice repetition towards neutral?

Thank you. We performed a new analysis and added the following text to the Results (lines 128-131):

> "Qualitatively, we observed the same pattern after correct or incorrect decisions (**Figure S2C,D**), and there was no significant difference between the drug effect on repetition probability after correct and error trials (t = 0.10, p = 0.925, BF = 0.239)."

One possible explanation for the finding that the drug effect on repetition probability was significant after correct choices but not errors is that participants performed at ~75% correct (see also **Figure 2A**), which results in a significantly smaller trial count for previous error trials, and therefore noisier estimates.

4. Related to the previous point, pupil size was recorded in the current study (Fig. 1D). This seems like a fantastic opportunity to compare effects of pupil size during the placebo sessions with that of atomoxetine during drug sessions. If the authors find evidence that pupil size promotes choice alternation, this within-participant difference to the effect of atomoxetine would provide an even more convincing basis for the discussion of the effects of tonic vs. phasic catecholamine levels.

Thank you for this great suggestion, which we have now followed with detailed analyses of the pupil data. Indeed, we show that overall pupil size is affected by atomoxetine (**Figure 1D**), and it is possible that the same holds for evoked pupil responses. But we could exploit our within-subject design to assess the effects on history bias of the evoked pupil responses measured specifically during the placebo sessions, which we think is the cleanest way of doing this. In short, this showed that (i) task-evoked pupil responses during placebo predicted a reduction of repetition probability, mimicking the effect of atomoxetine (**Figure 4B)**, and (ii) across-subjects, the drug effect on pupil size predicted the drug effect on repetition probability (**Figure 4C**). These new results are described in lines 207-240. Please see our reply to R1 point 1. Thank you again for pushing us on this point.

5. I was wondering how P(repeat) was calculated? The authors write: "Repetition probability was defined as the fraction of choices that was the same as the choice on the previous trial." Does this mean P(repeat) = P(Response(t) = Response(t-1))? Or is P(repeat) the average between p(Response(t) =

Left | Response(t-1) = Left) and p(Response(t) = Right | Response(t-1) = Right)? If I'm not mistaken, the former definition would include history-independent side biases in P(repeat). I would appreciate if this would be described in more detail.

P(repeat) was originally computed as P(Response(t) = Response(t-1)). However, we agree with you that controlling for potential side biases is important, and we have now done so throughout in the way suggested by the reviewer. We now write in the Results (lines 121-124):

> "We operationalized these time-varying prior expectations as the "repetition probability", the probability of repeating the choice from the previous trial, irrespective of a potential overall bias for the left or right choice options (Methods)."

This change made our results even stronger (**Figure 2D-F**). Thank you for pointing us to this.

6. For the computational interpretation, I am wondering whether one can distinguish down-weighting of prior expectations from weaker updating of priors. Especially since catecholamines have been implicated in modulating learning rates, it seems possible that reduced history biases stem from a weaker updating of priors rather than down-regulating the impact of priors. Is this a distinction the authors would like to make? I think it would be worthwhile to discuss this in more detail.

Thank you for this interesting suggestion. We believe that our current approach is simply not suited to distinguish between these scenarios: weaker updating of priors versus down-regulating the impact of priors. Doing so would require decomposing the updating step, using a richer behavioral task and model, and ideally also in terms of neural dynamics[14]. Since this requires detailed assessment of neural data, this is beyond the scope of the current study.

7. P.8, line 348: "Reaction time (RT) was defined as the time from stimulus offset until the button press." Should this be "stimulus onset"?

Thank you; we fixed this typo.

8. P.9, line 387: The authors write "pupil predicted shift". I think this should say "Atomoxetine predicted shift".

Thank you; we fixed this typo.

## References


1. Pfeffer, T. *et al.* Catecholamines alter the intrinsic variability of cortical population activity and perception. *PLOS Biology* **16**, e2003453 (2018).
2. Pfeffer, T. *et al.* Circuit mechanisms for the chemical modulation of cortex-wide network interactions and behavioral variability. *Sci Adv* **7**, eabf5620 (2021).
3. Nuiten, S. A., de Gee, J. W., Zantvoord, J. B., Fahrenfort, J. J. & van Gaal, S. Catecholaminergic neuromodulation and selective attention jointly shape perceptual decision-making. *Elife* **12**, RP87022 (2023).
4. Nuiten, S. A., Gee, J. W. de, Zantvoord, J. B., Fahrenfort, J. J. & Gaal, S. van. Pharmacological Elevation of Catecholamine Levels Improves Perceptual Decisions, But Not Metacognitive Insight. *eNeuro* **11**, (2024).
5. Joshi, S. & Gold, J. I. Pupil Size as a Window on Neural Substrates of Cognition. *Trends in Cognitive Sciences* (2020) doi:10.1016/j.tics.2020.03.005.
6. Reimer, J. *et al.* Pupil fluctuations track rapid changes in adrenergic and cholinergic activity in cortex. *Nat Commun* **7**, 13289 (2016).
7. Joshi, S., Li, Y., Kalwani, R. M. & Gold, J. I. Relationships between Pupil Diameter and Neuronal Activity in the Locus Coeruleus, Colliculi, and Cingulate Cortex. *Neuron* **89**, 221–234 (2016).
8. Breton-Provencher, V. & Sur, M. Active control of arousal by a locus coeruleus GABAergic circuit. *Nat Neurosci* **22**, 218–228 (2019).
9. Maheu, M., Donner, T. H. & Wiegert, J. S. Serotonergic and Noradrenergic Interactions in Pupil-linked Arousal. 2025.03.26.644382 Preprint at https://doi.org/10.1101/2025.03.26.644382 (2025).
10. Yu, A. J. & Dayan, P. Uncertainty, neuromodulation, and attention. *Neuron* **46**, 681–692 (2005).
11. Akaishi, R., Umeda, K., Nagase, A. & Sakai, K. Autonomous Mechanism of Internal Choice Estimate Underlies Decision Inertia. *Neuron* **81**, 195–206 (2014).
12. Urai, A. E., Braun, A. & Donner, T. H. Pupil-linked arousal is driven by decision uncertainty and alters serial choice bias. *Nat Commun* **8**, 14637 (2017).

13. Urai, A. E., de Gee, J. W., Tsetsos, K. & Donner, T. H. Choice history biases subsequent evidence accumulation. *eLife* **8**, e46331 (2019).
14. Murphy, P. R., Wilming, N., Hernandez-Bocanegra, D. C., Prat-Ortega, G. & Donner, T. H. Adaptive circuit dynamics across human cortex during evidence accumulation in changing environments. *Nat Neurosci* **24**, 987–997 (2021).
