# Supplementary material for: Catecholamines reduce choice history biases in perceptual decision making
Source: PLoS Biol. 2025 Sep 3;23(9):e3003361. doi: 10.1371/journal.pbio.3003361 (PMC12407465; doi:10.1371/journal.pbio.3003361)
Supplement: Attachment — Submitted filename: deGeeEtAlRebuttal_final.docx [file pbio.3003361.s008.docx]

REVIEWS:

Reviewer remarks:

Reviewer #1 (Rei Akaishi, signed his report): The authors have adequately addressed the concerns raised in my previous review.

Thanks again for reviewing our paper and your constructive comments.

However, I note that the mechanisms underlying catecholamine effects on choice repetition may be more complex than what can be fully captured by the current experimental design. In particular, future investigations should carefully examine the implications of the Yu-Dayan theoretical framework for understanding choice repetition bias. Such studies would also contribute to a more nuanced understanding of the underlying mechanisms driving choice repetition phenomena.

Completely agreed. Upon further suggestion by the editor, we have changed the title to: “Catecholamines reduce choice history biases in perceptual decision making”.

Reviewer #2: The authors have carefully addressed my points and I have no further issues.

Thanks again for reviewing our paper and your constructive comments.

Minor comment:

I think on page 4, line 143, the authors mean to refer to Figure 2E, not S2E.

Thank you for spotting this!
